# Auricular acupuncture for premature ovarian insufficiency: A protocol for systematic review and meta-analysis

**DOI:** 10.1097/MD.0000000000022212

**Published:** 2020-09-25

**Authors:** Yehao Luo, Donghan Xu, Xiusong Tang, Luqiu Wei, Lizhen Wang, Yuzhou Pang, Gang Fang

**Affiliations:** a Guangxi university of Traditional Chinese Medicine, Nanning, Guangxi Province; b Macau University of Science and Technology, Macau; c Hospital of Chengdu University of Traditional Chinese Medicine, Chengdu, Sichuan Province; d Guangxi Zhuang Yao Medicine Center of Engineering and Technology, Guangxi University of Chinese Medicinee, Nanning, Guangxi Province, China.

**Keywords:** auricular acupuncture, premature ovarian insufficiency, protocol, systematic review

## Abstract

**Background:**

A lot of attention has been given to premature ovarian insufficiency (POI) as it poses considerable health risks to women. It is characterized by oligomenorrhea, amenorrhea, infertility, autoimmune disorders, and ischemic heart disease, with increased mortality. Previous research indicates that auricular acupuncture is proven effective in treating POI in clinical practice. However, systematic review has not been carried out. Therefore, this study aims at evaluating the curative effect and safety of auricular acupuncture treatment for POI through systematic review and meta-analysis.

**Methods and analysis:**

The following databases will be searched for relevant information before August 2020: PubMed, Embase, Cochrane Library, Web of Science, and CNKI. Major results: levels of follicle-stimulating hormone (FSH), luteinizing hormone (LH), and estrogen (E2). Secondary results: modified Kupperman Index, imaging results including ovarian size, antral follicle count, and blood flow changes in the ovary using color Doppler ultrasound; total effective rate, adverse event and intervention, and hospitalization expenses. Data will be collected independently by 2 researchers, and the risk of bias in meta-analysis will be evaluated according to “Cochrane Handbook for Systematic Reviews of Interventions”. All data analysis will be conducted using Review Manager V.5.3. and Stata V.12.0.

**Results:**

The curative effect and safety of auricular acupuncture treatment for POI patients will be evaluated systematically.

**Conclusion:**

In the systematic review, the published evidence of auricular acupuncture treatment for POI will be summarized to provide guidance for promotion and application.

**Ethics and dissemination:**

The private information from individuals will not be published. This systematic review also will not involve endangering participant rights. Ethical approval is not required. The results may be published in a peer-reviewed journal or disseminated in relevant conferences.

Open Science Framework (OSF) registration number: http://osf.io/tg9mw

## 1 Introduction

Premature ovarian insufficiency (POI), previously referred to as premature ovarian failure or premature menopause, is a common disease with the global incidence of 1% to 3%. Its most significant clinical manifestations include dysfunction of ovaries, amenorrhea and infertility before age 40.^[1]^ Twenty five percent to 30% of POI cases are caused by known hereditary causes, but the cause of 50% to 90% of POF cases is unknown (idiopathic).^[2]^ Currently, there is no therapeutic intervention that is able to effectively recover the fertility of POI patients, and this severely affects the reproduction and life quality of women of childbearing age.^[3]^

Currently, hormone replacement therapy (HRT) is used as a major approach to treat POI in Western Medicine,^[4]^ and stem cell transplantation is developing.^[5]^ A great deal of clinical and research has indicated that it has definite therapeutic effect, high safety and high patient acceptance to treat POI using auricular acupuncture alone or in combination with traditional Chinese medicine or Western medicine, however the mechanism of action is not clear.^[6]^ Auricular acupuncture is a traditional Chinese collateral meridian therapy, including auricular embedding therapy, auricular plaster therapy, bloodletting at auricular points and automatic auricular therapeutic device. Clinical research has indicated that auricular acupuncture is a simple, inexpensive, and manageable non-pharmaceutical therapy.^[7]^ Previous reviews have indicated the result of auricular acupuncture treatment for POI.^[8,9]^ Compared with HRT, it is a simple and safe therapy for POI that is able to significantly improve symptoms.^[10]^ However, there is a lack of evidence of results of auricular acupuncture in treating POI. Therefore, the paper will evaluate the effectiveness and safety of auricular acupuncture treatment for POI. This review will be the first evaluation of the impact of auricular acupuncture.

## 2 Objectives

In a randomized controlled trial (RCT), the efficacy and side effects of auricular acupuncture in treating POI have been evaluated systematically. We expect to provide reference for POI treatment in the field of traditional Chinese medicine.

## 3 Methods

### 3.1 Study registration

The protocol of the systematic review has been registered.

Registration: OSF Preregisration.2020, Aug.14. osf.io/tg9 mw. This systematic review protocol will be conducted and reported strictly according to Preferred Reporting Items for Systematic Reviews and Meta-Analyses (PRISMA) statement guidelines, and the important protocol amendments will be documented in the full review.

### 3.2 Criteria for considering studies for this review

We will strictly screen studies that meet the following inclusion criteria.

#### 3.2.1 Type of included studies

Only RCTs (except Quasi-RCTs and cluster RCTs) will be included. Animal mechanism studies and non-randomized clinical trials will be excluded. Article that substantially overlaps with another published article in print or electronic media will be excluded. Duplicate publications produced by a single experiment and published as separate papers with different criteria for.

Measuring results, priority will be given to original publications and other publications will be excluded. The language and time of publication will not be restricted.

#### 3.2.2 Participants

We will include RCTs of participants of 18 years or older, of any sex, race/ethnicity, and diagnosed with POI (diagnosis as defined by the individual trial). We will accept RCTs in which participants had any duration and severity of the disease. Due to different pathways and mechanisms, we will exclude the trials of patients with POF caused by ovariectomy, congenital developmental dysplasia of reproductive organ, acquired organic diseases (e.g., malignant tumors of reproductive organ), endocrine system diseases, iatrogenic injuries (e.g., surgery, chemoradiotherapy).

#### 3.2.3 Interventions and controls

The interventions included auricular point alone or in combination with other conventional treatments. The control group received only conventional treatment. The choice of conventional treatment for each RCT may not be entirely consistent, but auricular point alone or in combination with conventional treatment should be the only difference between intervention and control.

#### 3.2.4 Type of outcome measures

Major results: levels of follicle-stimulating hormone (FSH), luteinizing hormone (LH), and estrogen (E2). Secondary results: modified Kupperman Index, imaging results including ovarian size, antral follicle count and blood flow changes in the ovary using color Doppler ultrasound; total effective rate, adverse event and intervention, and hospitalization expenses.

### 3.3 Search methods

#### 3.3.1 Search resources

**Figure 1:**
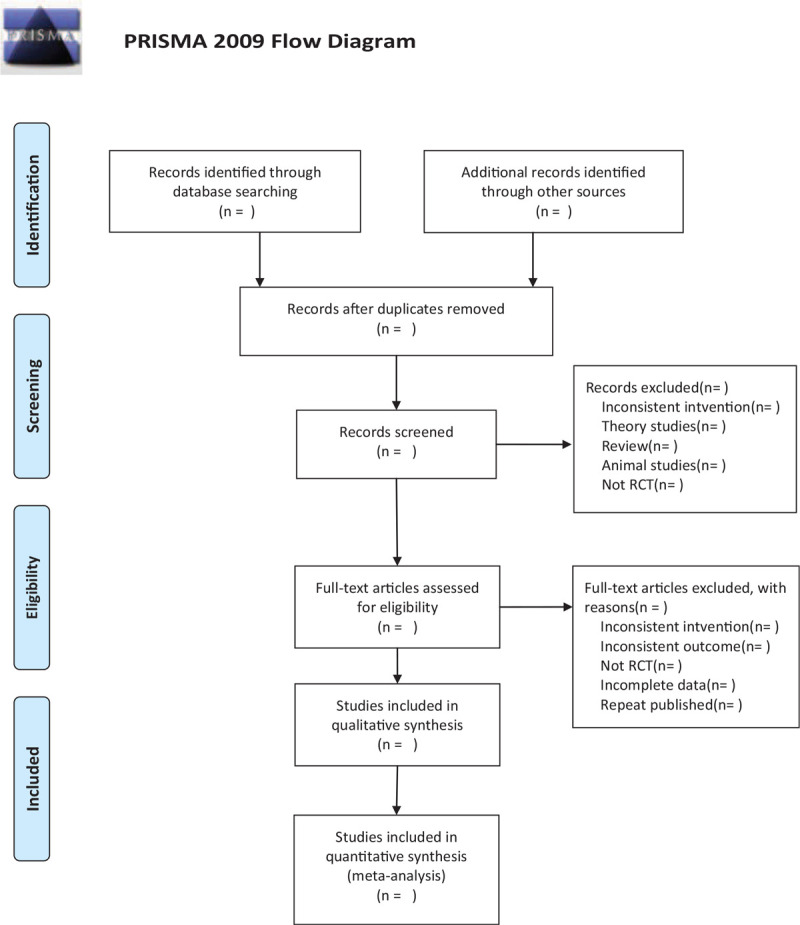
The research flowchart. This figure shows the Identification, Screening, Eligibility and Included when we searching articles.

This review will include the following electronic databases from their inception to Aug 2020: Electronic database includes PubMed, Embase, Cochrane Library, Web of Science, CNKI (Fig. 1.) The research flowchart.

#### 3.3.2 Search strategies

The following MeSH terms and their combinations will be searched:

1. auricular acupuncture OR auricular needle OR ear acupuncture OR auricular point sticking OR auricular point;
2. RCT OR RCTs;
3. premature ovarian failure OR premature ovarian insufficiency OR primary ovarian insufficiency OR decreased ovarian reserve function.

The search strategy for PubMed is shown in (Table 1). Other electronic data bases will be used the same strategy.

**Table 1:**
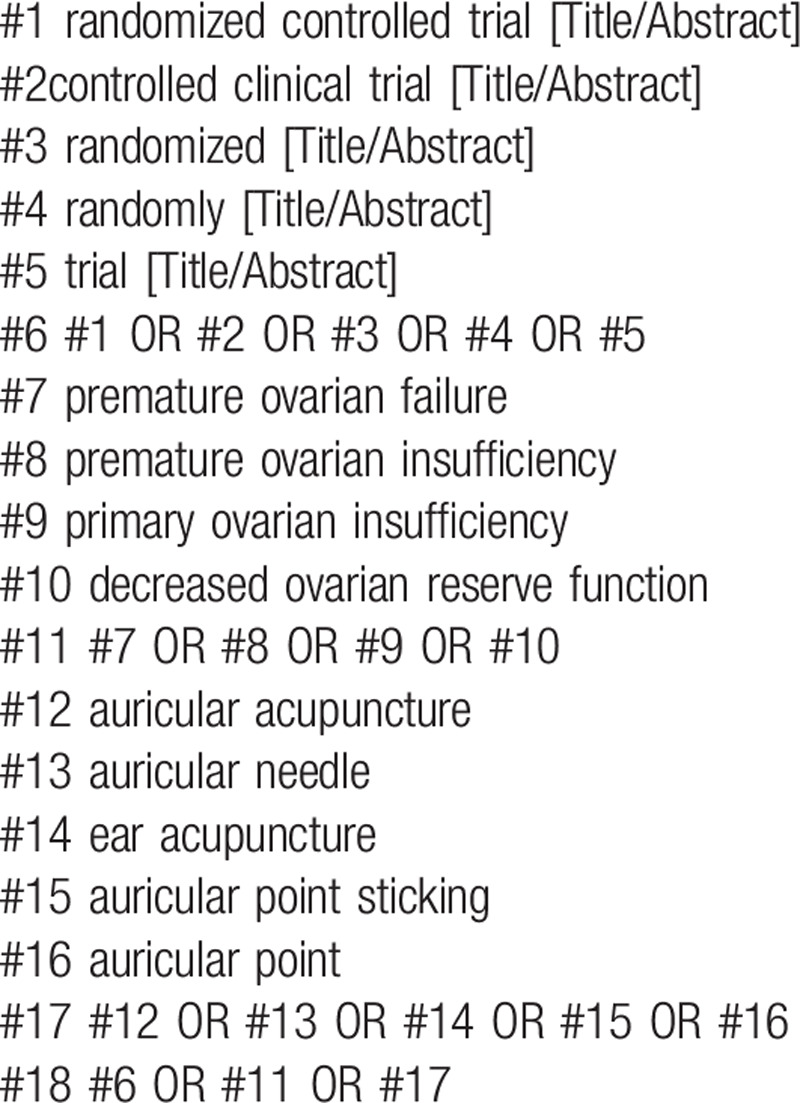
Search strategy in PubMed database.

### 3.4 Data collection and analysis

#### 3.4.1 Studies selection

There will be 2 researchers (YL and DX) carry out the selection of research literature independently using endnote x^9^ software. We will first make the preliminary selection by screening titles and abstracts. Secondly, we will download full text of the relevant studies for further selection according to the inclusion criteria. If there is any different opinion, 2 researchers will discuss and reach an agreement. If a consensus could not be reached, there will be a third researcher (FG) who make the final decision. The details of selection process will be displayed in the PRISMA flow chart.

#### 3.4.2 Assessment of risk of bias

The assessment of risk of bias will be carried out by 2 independent reviewers (LYH and WLQ), using the Cochrane Collaborations “Risk of bias” tool. Study bias will be conducted as either: “unclear,” “low,” or “high” risk for the following criteria: random sequence generation, allocation concealment, blinding, incomplete data, selective outcome reporting, and other bias. The assessment of the bias has caused controversy, there is a need for discussion with a third reviewer (PYZ). The graphic representations of potential bias within and across studies using Rev Man V.5.3.5.

#### 3.4.3 Measures of treatment effect

Statistical analyses will be conducted using the risk ratio with 95% confidence intervals (CIs). Odds ratio (OR) and relative risk (RR) are commonly used for dichotomous outcomes data. For continuous outcomes, the weighted mean difference (WMD), or the standard mean difference (SMD) will be analyzed.

#### 3.4.4 Unit of analysis issues

The unit of analysis will be the individual participant.

#### 3.4.5 Dealing with missing data

Among the results of several studies with insufficient data or missing data, the corresponding author will be contacted to complement the contents. If the corresponding author cannot be contacted, the data alone will be conducted.

#### 3.4.6 Assessment of heterogeneity

The assessment of heterogeneity will be conducted by Review Manager (V.5.3.5). Chi-Squared test and *I*^2^value of the forest, plot will be calculated to assess heterogeneity, according to the Cochrane Handbook. The *I*^2^value is classified into 4 levels: little or no heterogeneity (0%–40%), moderate heterogeneity (30%–60%), substantial heterogeneity (50%–90%), and considerable heterogeneity (75%–100%).

#### 3.4.7 Assessment of reporting biases

If the numbers of available studies are sufficient, funnel plots will be assessed reporting biases.

#### 3.4.8 Data synthesis

Review Manager (V.5.3.5) will be used to analyze. The test indicated little or no heterogeneity; a fixed effect model will be used for data. The random effect model will be adopted when there is considerable heterogeneity (I2% ≥ 50%). If there is considerable variation in results (I2% ≥ 75%), the meta-analysis will not be performed. The narrative and qualitative summary will be available.

#### 3.4.9 Subgroup analysis and investigation of heterogeneity

Subgroup analysis will be conducted to assess heterogeneity. The different types of auricular acupuncture (embedding therapy on-ear point, auricular point seeds pressure, bloodletting at auricular points, auricular point on auto chemotherapy) may be affected heterogeneity.

#### 3.4.10 Sensitivity analysis

Sensitivity analysis will be used to assess the robustness of the results. It is possible to determine according to methodological quality, sample size, and analysis-related issues. The studies that follow a sequence will be removed from all the inclusion reviews. The Chi-Squared test and *I*^2^ value will be used to quantify statistical heterogeneity.

#### 3.4.11 Summary of evidence

The assessment of evidence for all outcomes will be summarized using the Grading of Recommendations Assessment, Development and Evaluation (GRADE) approach. The quality of evidence will be rated as high, moderate, low, and very low quality.

## 4 Discussion

Premature ovarian insufficiency has many causes, including genetic, immune and metabolic factors, and enzyme defect. They can cause premature ovarian insufficiency by reducing follicles in the follicular pool or follicular dysfunction.^[11,12]^ From the perspective of channels and collaterals, all channels and collaterals converge at ears, and auricular points are distributed like an upside-down infant. By stimulating particular auricular points, the physiological functions of particular parts of the body can be strengthened, the circulation of qi and blood can be promoted, and the reproductive function of hypothalamus-pituitary-ovary axis can be regulated.^[13]^ According to research on clinical treatment, it has good therapeutic effect to treat POI using auricular points. As POF is in the final stage of ovarian function failure, treatment is often delayed when the disease is diagnosed. Therefore in 2016, “premature ovarian failure” was replaced by “premature ovarian failure” in “Guideline on Management of Women with Premature Ovarian Insufficiency” by ESHRE. Early detection and safe treatment with lasting therapeutic effect are crucial. Different from HRT, auricular acupuncture is not hormone but it has hormone-like effects. The goal of recovering ovarian function can be achieved by overall regulation of patients hormone levels. Its advantages include simple operation, high safety and reliability, lasting therapeutic effect and high treatment acceptance. It has prominent advantages in reducing complications of POI and relieving clinical symptoms of patients with POI, including irregular menstruation, infertility, menopausal syndrome and hormonal regulation. However, the mechanism and standards of treating POI using auricular points are not expounded systematically. In short, this systematic review and meta-analysis can help identify the potential value of auricular points in treating POI and improving amenorrhea, infertility and life quality. This study can provide a foundation for the release of POI treatment guidelines and treatment options of POI patients, and thus benefit more patients.

## Author contributions

**Conceptualization:** Yehao Luo, Donghan Xu.

**Data curation:** Donghan Xu, Luqiu Wei, Fang Gang.

**Formal analysis:** Yehao Luo, Xiusong Tang, Lizhen Wang.

**Funding acquisition:** Yuzhou Pang, Fang Gang.

**Investigation:** Donghan Xu, Luqiu Wei.

**Project administration:** Yuzhou Pang.

**Quality assessment:** Fang Gang, Yehao Luo.

**Software:** Luqiu Wei, Donghan Xu, Lizhen Wang.

**Supervision:** Yehao Luo.

**Validation:** Yehao Luo, Lizhen Wang.

**Writing – original draft:** Yehao Luo, Donghan Xu, Xiusong Tang.

**Writing – review & editing:** Luqiu Wei, Fang Gang, Yuzhou Pang.
